# Low-flow CO_2_ removal in combination with renal replacement therapy effectively reduces ventilation requirements in hypercapnic patients: a pilot study

**DOI:** 10.1186/s13613-019-0480-4

**Published:** 2019-01-07

**Authors:** Jens Nentwich, Dominic Wichmann, Stefan Kluge, Simone Lindau, Haitham Mutlak, Stefan John

**Affiliations:** 1 Medical Intensive Care, Department of Cardiology, Klinikum Nuremberg, Paracelsus Medical University, Nuremberg, Germany; 2 Department of Intensive Care Medicine, University Medical Center Hamburg-Eppendorf, Hamburg, Germany; 3 Department of Anesthesia, Intensive Care Medicine and Pain Therapy, University Hospital Frankfurt, Frankfurt, Germany

**Keywords:** Low-flow extracorporeal CO_2_ removal, Mechanical ventilation, Lung protection, Ventilator-induced lung injury, Acute kidney injury, Renal replacement therapy, Multi-organ support

## Abstract

**Background:**

Lung-protective strategies are the cornerstone of mechanical ventilation in critically ill patients with both ARDS and other disorders. Extracorporeal CO_2_ removal (ECCO_2_R) may enhance lung protection by allowing even further reductions in tidal volumes and is effective in low-flow settings commonly used for renal replacement therapy. In this study, we describe for the first time the effects of a labeled and certified system combining ECCO_2_R and renal replacement therapy on pulmonary stress and strain in hypercapnic patients with renal failure.

**Methods:**

Twenty patients were treated with the combined system which incorporates a membrane lung (0.32 m^2^) in a conventional renal replacement circuit. After changes in blood gases under ECCO_2_R were recorded, baseline hypercapnia was reestablished and the impact on ventilation parameters such as tidal volume and driving pressure was recorded.

**Results:**

The system delivered ECCO_2_R at rate of 43.4 ± 14.1 ml/min, PaCO_2_ decreased from 68.3 ± 11.8 to 61.8 ± 11.5 mmHg (*p* < 0.05) and pH increased from 7.18 ± 0.09 to 7.22 ± 0.08 (*p* < 0.05). There was a significant reduction in ventilation requirements with a decrease in tidal volume from 6.2 ± 0.9 to 5.4 ± 1.1 ml/kg PBW (*p* < 0.05) corresponding to a decrease in plateau pressure from 30.6 ± 4.6 to 27.7 ± 4.1 cmH_2_O (*p* < 0.05) and a decrease in driving pressure from 18.3 ± 4.3 to 15.6 ± 3.9 cmH_2_O (*p* < 0.05), indicating reduced pulmonary stress and strain. No complications related to the procedure were observed.

**Conclusions:**

The investigated low-flow ECCO_2_R and renal replacement system can ameliorate respiratory acidosis and decrease ventilation requirements in hypercapnic patients with concomitant renal failure.

*Trial registration* NCT02590575, registered 10/23/2015.

## Background

Lung-protective strategies are the mainstay of mechanical ventilation in patients with ARDS and other inflammatory pulmonary disorders, as the use of lower tidal volumes and plateau pressures improves survival rates by reducing pulmonary stress and strain [1, 2]. In addition, there is a growing body of evidence that a further reduction in the mechanical stress resulting from positive pressure ventilation by further decreasing tidal volumes may be even more “lung protective” [3–5]. Whereas low tidal volume ventilation has been shown to reduce pulmonary inflammation and consequently mortality, it is often accompanied by hypercapnic acidosis, which will even be more pronounced under “ultra-protective” ventilation strategies. Although elevated PaCO_2_ levels in this setting (“permissive hypercapnia”) are often well tolerated and deemed to be safe [1–3], the degree to which hypercapnia may be tolerable or even beneficial by directly supporting anti-inflammation remains unclear [6–9]. Very recent data have shown a positive correlation between hypercapnic acidosis and mortality [10, 11], thereby casting doubt on the uncritical toleration of hypercapnic acidosis under lung-protective mechanical ventilation. On the other hand, many patients with ARDS present with multi-organ failure, e.g., due to septic shock, and consequently exhibit massive metabolic acidosis in combination with severe cardiovascular instability. This may further limit the concept of permissive hypercapnia since an additional decrease in pH may be considered unsafe in such patients. This problem is even more pronounced when aiming for an additional reduction in the invasiveness of mechanical ventilation by further reducing tidal volumes.

Although there are no conclusive data showing a reduction in mortality, there is evidence that extracorporeal CO_2_ removal (ECCO_2_R) can effectively normalize severe hypercapnia and facilitate ultra-protective ventilation strategies [12–15]. Whereas extracorporeal membrane oxygenation (ECMO) is becoming more and more widespread in the therapy of patients with severe ARDS and provides total decarboxylation in addition to oxygenation, such high-flow systems require substantial resources and carry a considerable risk of complications and are therefore limited to patients with severe hypoxemia [16–22]. Total extracorporeal CO_2_ removal can be achieved with less invasive techniques such as pumpless extracorporeal lung assist (pECLA) and other mid-flow systems [23, 24], but these techniques still require specialized vascular access and are therefore invasive and expensive.

On the other hand, it has repeatedly been demonstrated that even low-flow systems adapted from conventional renal replacement platforms with blood flow rates under 500 ml/min can achieve significant CO_2_ elimination (“respiratory dialysis”) [25–27]. Extracorporeal CO_2_ removal based on renal replacement platforms may be especially useful in mechanically ventilated patients with multi-organ failure, since in one- to two-thirds of those patients there is an indication for renal replacement therapy [28, 29]. Furthermore, concomitant lung and kidney injury may exhibit significant detrimental interaction (“organ cross talk”) [30, 31], which may negatively affect outcome. Because no additional vascular access other than the dialysis catheter is required, the implementation of a hollow-fiber gas exchanger in the renal replacement circuit could be an attractive therapeutic option in such patients.

Though the use of such combinations of ECCO_2_R and continuous renal replacement therapy (CRRT) has been reported [27, 32, 33], until recently no certified combination therapy has been available. In this pilot study, we describe for the first time the effectiveness of a commercially available combination of ECCO_2_R and CRRT regarding decarboxylation and ventilation as well as other clinical parameters.

## Methods

### Aims of the study, inclusion and exclusion criteria

The main goals of this multicenter observational pilot study were (1) to determine the changes in blood gases under the combined renal replacement and decarboxylation therapy and (2) to record the reduction in plateau pressures and tidal volumes that can thus be achieved. Secondary measurements included changes in ventilator settings, systemic hemodynamics, membrane lung performance, running time and patency of the extracorporeal circuit as well as documentation of system-related complications. The study protocol was approved by the local ethics committees at all three participating centers. We aimed to analyze data from 20 critically ill patients with the following inclusion criteria: (1) mechanical ventilation according to ARDS Network criteria with a prospected duration of at least 24 h, (2) hypercapnic acidosis with a pH below 7.30 and a PaCO_2_ of at least 55 mmHg under a plateau pressure of at least 25 cmH_2_O and (3) indication for renal replacement therapy. The study was registered under NCT02590575.

### Extracorporeal circuit

The Prismalung™ system (Baxter Gambro Renal, USA) consists of a 0.32-m^2^ membrane oxygenator that can be included in the Prismaflex™ organ support platform either in a stand-alone fashion or in combination with CRRT to provide low-flow CO_2_ removal. For this study, we utilized the combination therapy where the membrane oxygenator is inserted serially in the extracorporeal circuit downstream to the hemofilter. 100% oxygen was used as sweep gas. The Prismaflex™ system is equipped with a software extension; otherwise, there are no changes compared to the basic CRRT mode. Systemic anticoagulation with unfractioned heparin and a target aPTT (activated partial thromboplastin time) of 60 s was used to prevent clotting in the extracorporeal circuit. Temperature management was according to local practice standards using tube heating or optionally a conventional heat exchanger directly connected to the membrane oxygenator. Renal replacement therapy was provided according to local practice standards as continuous veno-venous hemofiltration (CVVHF) using bicarbonate-buffered replacement fluids. CRRT dose was calculated according to international guideline recommendations; approximately one-third of the dose was applied as pre-dilution.

### Study protocol

Patients underwent a checklist-based screening for inclusion criteria. If inclusion criteria were met and no exclusion criteria were present, informed consent was obtained by the patient or legal guardian. After inclusion patients underwent a 2-h stabilization period to ensure stable cardiovascular and respiratory conditions before implementation of the extracorporeal circuit. Adequate analgosedation with a Richmond agitation and sedation scale (RASS) target of − 4 was provided, neuromuscular blockade was not mandatory. During this period, vascular access was obtained with a conventional 13.5 French Shaldon catheter (Bard Access Systems, USA) via an internal jugular (75% of patients, catheter length 20 cm) or femoral vein (25% of patients, catheter length 24 cm) and the system was primed. The stabilization period could be shortened if the immediate commencement of renal replacement therapy was deemed to be necessary. After connecting the patient to the circuit and before starting sweep gas flow, baseline parameters were collected. Then sweep gas was started at a flow rate of 8 l/min and remained unchanged throughout the study. After recording changes in blood gases after a running time of 30 min, ventilator settings were adapted (i.e., *P*_plat_ lowered) with the goal of reestablishing baseline PaCO_2_. Original PaCO_2_ was reinstated using end-tidal CO_2_ as guidance and confirmed through blood gas analysis. After another 30 min data were collected and again at 24, 48 and 72 h after implementation. Ventilator settings were left to the discretion of the treating physician as soon as the initial data collections (at 30 and 60 min) were completed. The study ended after 72 h or loss of the system due to clotting. In case of system loss within the first 24 h, a new system could be implemented and data collection continued.

### Statistical analysis and ECCO_2_R calculation

Data were collected using paper-based case record forms, and a database was created with conventional spreadsheet software (Microsoft Excel 2010). Mean and median values as well as standard deviations were calculated and one-sided Student’s *t* test was used for statistical comparison assuming normally distributed data. Diagrams were created with SciDAVis open-source software (version 1.22). Blood gas analysis was performed using a conventional blood gas analyzer (ABL 800 Flex, Radiometer, Denmark), and ECCO_2_R rate was calculated from blood flow and the difference between blood CO_2_ content at the beginning and end of the extracorporeal circuit according to the following equation [34]:

CO_2_ removal rate = (CO_2_ arterial content−CO_2_ venous content) × blood flow = 24 × ((HCO_3_ arterial + 0.03 × PCO_2_ arterial)−(HCO_3_ venous + 0.03 × PCO_2_ venous)) × blood flow with arterial and venous referring to the arterial and venous lines of the extracorporeal circuit, respectively.

## Results

### Patient characteristics

Between January 2016 and February 2017, 26 critically ill patients in three centers were included in the study. Because of the wrong application of inclusion criteria and inconsistent baseline measurements, data of 20 patients were included in the final analysis. All patients were on pressure-controlled mechanical ventilator support, received an opiate-based analgosedation regime and were on vasopressor support. The leading indications for renal replacement therapy were sepsis in 9/20 patients and established chronic renal failure in 6/20 patients; other indications included shock and acute cardiorenal syndrome. One surgical patient was included; all other patients (19/20) were medical. Patient characteristics and parameters at inclusion are given in Tables 1 and 2, respectively.

**Table 1:** Patient characteristics

Age (years)	64 (43–82)
Male sex	12/20 (60%)
BMI (kg/m^2^)	29.4 (24.2–39.7)
SAPS II	57 (27–79)
Patient category	
Medical	19/20 (95%)
Surgical	1/20 (5%)
SOFA	14 (8–18)
Main diagnoses	
Pneumonia	17/20 (85%)
Septic shock	14/20 (70%)
ARDS	8/20 (40%)
COPD	7/20 (35%)
CIHD	6/20 (30%)
Analgosedation	
Opiates	20/20 (100%)
Sedation	19/20 (95%)
NMBA	5/20 (25%)
Hemodynamic support	
Vasopressors	20/20 (100%)
Inotropes	2/20 (10%)

*BMI* body mass index; *SAPS* Simplified Acute Physiology Score; *SOFA* Sequential Organ Failure Assessment; *ARDS* acute respiratory distress syndrome; *COPD* chronic obstructive pulmonary disease; *CIHD* chronic intermittent hemodialysis; *NMBA* neuromuscular blocking agent

**Table 2:** Parameters at inclusion

Parameter	Value ± SD	Range
pH	7.20 ± 0.08	7.02–7.31
PaCO_2_ (mmHg)	66.3 ± 8.7	56.1–84.4
HCO_3_ (mmol/l)	24.1 ± 3.9	17.1–34.3
CaCO_2_ (ml/l)	626 ± 93	457–881
SBE (mmol/l)	− 2.4 ± 4.8	− 11.7–11.9
SaO_2_ (%)	95 ± 2	88–98
*P*/*F* (mmHg)	159 ± 36	107–224
*V*_T_/PBW (ml/kg)	6.0 ± 0.7	4.5–7.9
RR (bpm)	25 ± 4	16–31
RMV (l/min)	9.6 ± 1.7	6.3–12.7
*P*_plat_ (cmH_2_O)	30 ± 4	25–38
PEEP (cmH_2_O)	12 ± 3	6–18
ΔP (cmH_2_O)	18 ± 4	11–26
*C*_dyn_ (ml/cmH_2_O)	27.5 ± 10.8	14.9–48.0
HR (bpm)	101 ± 20	70–150
MAP (mmHg)	72 ± 12	56–98
Norepinephrine dose (mg/h]	2.7 ± 2.2	0.4–9.0
RASS	− 4 ± 1	− 5 to − 3

*PaCO*_*2*_ arterial CO_2_ partial pressure; *HCO*_*3*_ bicarbonate concentration; *CaCO*_*2*_ arterial CO_2_ content; *SBE* standard base excess; *SaO*_*2*_ arterial O_2_ saturation; *P*/*F* oxygenation index; *V*_T_ tidal volume; *PBW* predicted body weight; *RR* respiratory rate; *RMV* respiratory minute volume; *P*_plat_ plateau pressure; *PEEP* positive end-expiratory pressure; Δ*P* driving pressure; *C*_dyn_ dynamical compliance; *HR* heart rate; *MAP* mean arterial pressure; *RASS* Richmond agitation and sedation scale

### ECCO_2_R, blood gases and CO_2_ transfer

**Fig. 1:**
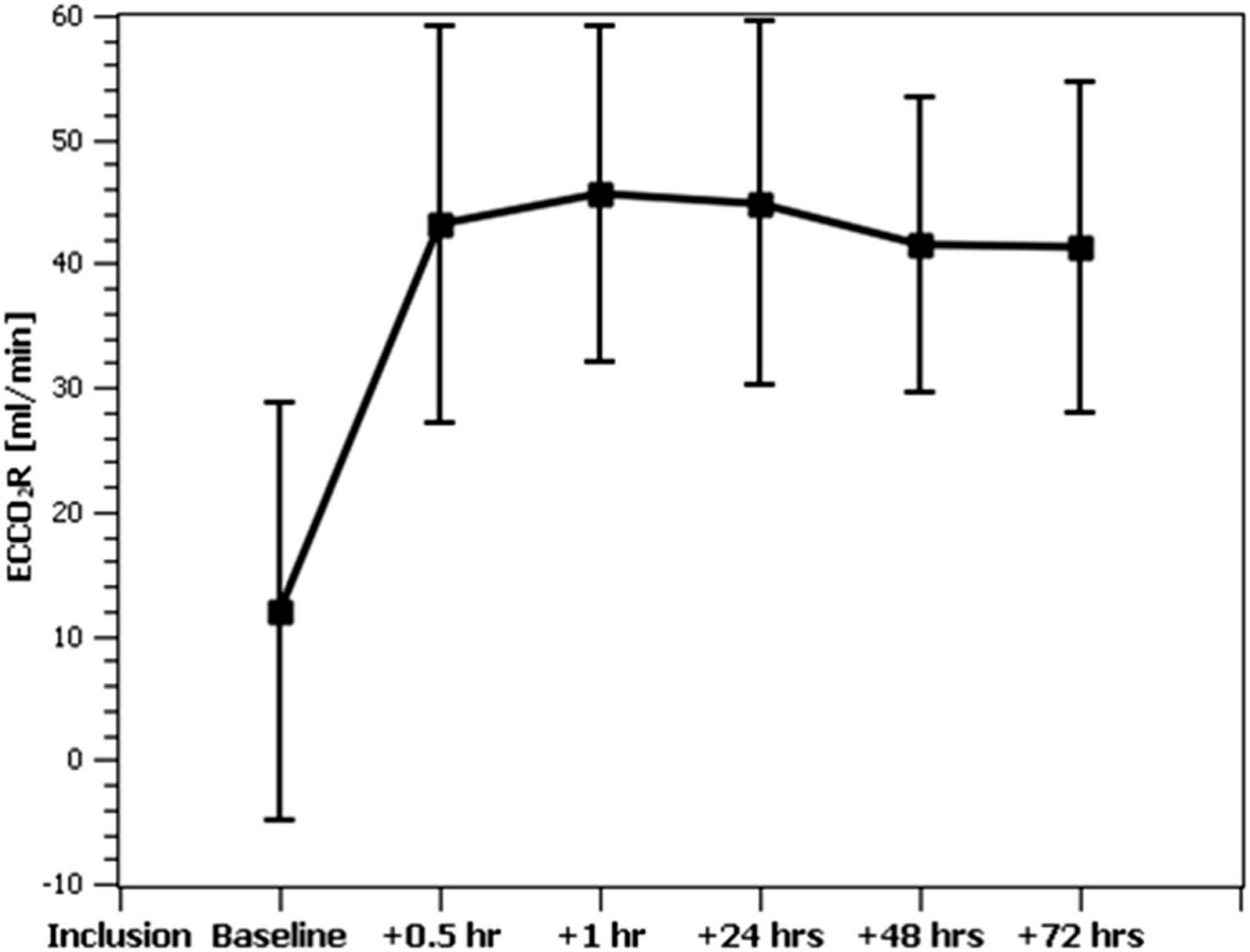
Time course of ECCO_2_R over the study period. At baseline, the patient was connected to the extracorporeal circuit with the sweep gas flow over the membrane lung turned off

**Fig. 2:**
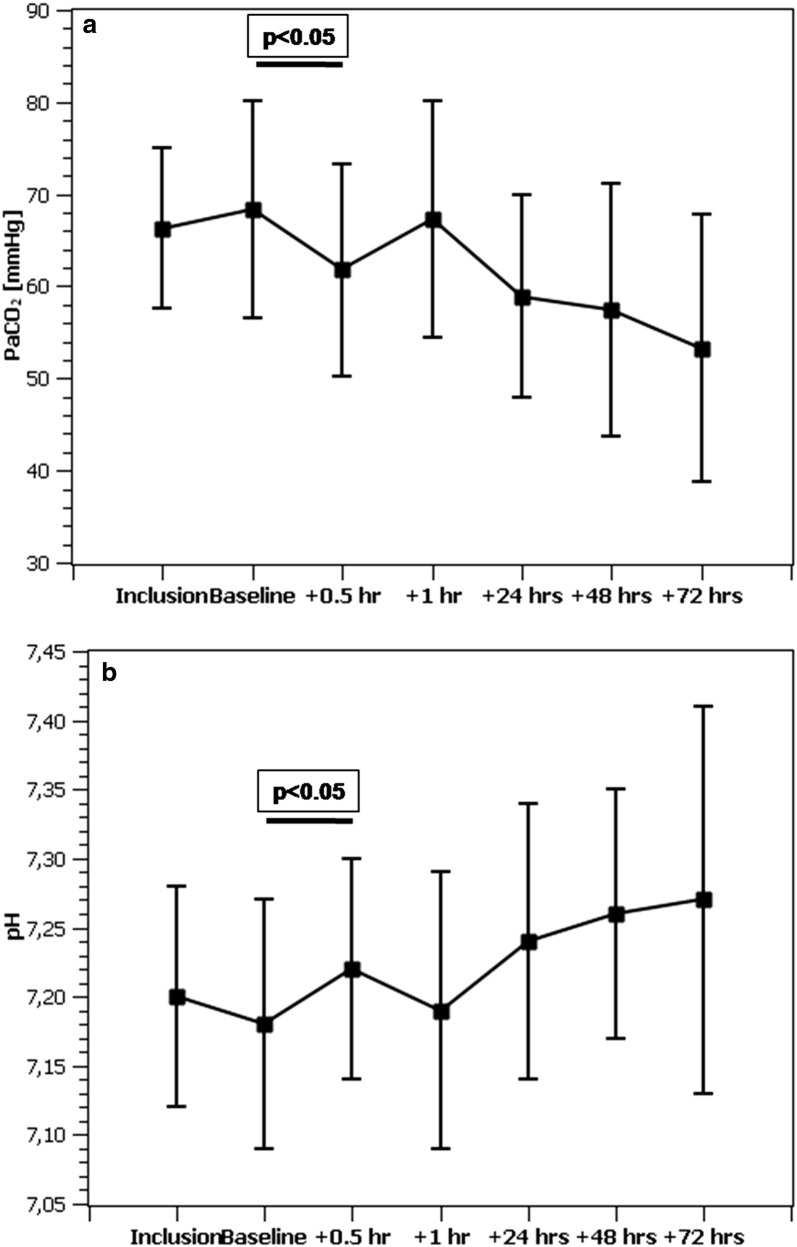
Time course of **a** arterial CO_2_ partial pressure and **b** pH over the study period. After adjusting ventilation parameters to reestablish baseline PaCO_2_ at 1 h, a trend toward further normalization of hypercapnic acidosis was observed

The average overall ECCO_2_R rate of the combined system was 43.4 ± 14.1 ml/min with a maximum of 75.0 ml/min and remained above 40 ml/min during the course of the study (Fig. 1). The CO_2_ elimination rate of the membrane lung alone was slightly higher (45.4 ± 15.7 ml/min). The resulting decrease in the CO_2_ content of extracorporeal blood was 113 ml/l (640 ± 98 vs. 527 ± 103 *p* < 0.05) corresponding to a decrease in patient arterial CO_2_ of 6.5 mmHg (68.3 ± 11.8 vs. 61.8 ± 11.5, *p* < 0.05) and an increase in pH from 7.18 ± 0.09 to 7.22 ± 0.08 (*p* < 0.05) after 30 min (Table 3). No change in systemic oxygenation was observed at this point. During the remaining study period, a further normalization of blood gases was observed (Fig. 2). Passage of the membrane lung reduced extracorporeal blood CO_2_ content from 645 ± 94 to 527 ± 103 ml/l (− 18%), corresponding to a drop in PCO_2_ from 66.6 ± 12.6 to 32.3 ± 5.5 mmHg (− 52%).

**Table 3:** Changes in arterial CO_2_ load, partial pressure and pH between baseline and at 0.5 h

	Baseline	+ 0.5 h	Δ (%)	*P*-value
pH	7.18 ± 0.09	7.22 ± 0.08	+ 0.04	< 0.05
PaCO_2_ (mmHg)	68.3 ± 11.8	61.8 ± 11.5	− 6.5 (− 9.5)	< 0.05
CaCO_2_(ml/l)	623 ± 106	611 ± 98	− 12 (− 1.9)	< 0.05

*PaCO*_*2*_ arterial CO_2_ partial pressure; *CaCO*_*2*_ arterial CO_2_ content

### Ventilation

**Fig. 3:**
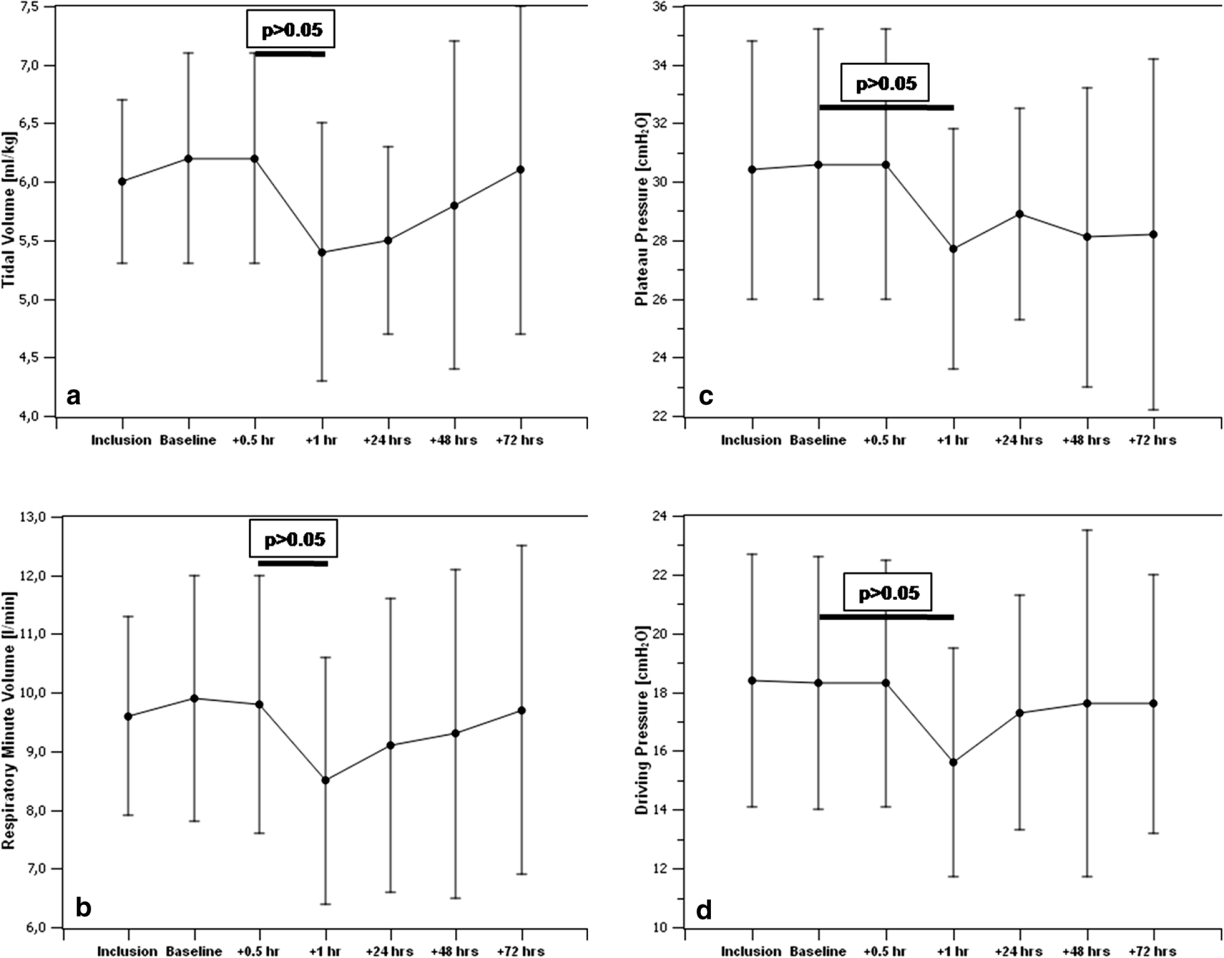
Changes in ventilation under ECCO_2_R over the study period showing a significant decrease in tidal volumes per predicted body weight (**a**) and respiratory minute ventilation (**b**) compared to baseline, corresponding to a significant decrease in plateau (**c**) and driving pressures (**d**)

After ventilator settings were adapted and baseline PaCO_2_ was reestablished, we recorded a decrease in respiratory minute ventilation from 9.9 ± 2.1 to 8.5 ± 2.1 l/min (− 1.4 l/min, *p* < 0.05), corresponding to a reduction in tidal volume from 6.2 ± 0.9 to 5.4 ± 1.1 ml/kg of predicted body weight (PBW) (− 0.8 ml/kg, *p* < 0.05). Plateau pressures decreased from 30.6 ± 4.6 to 27.7 ± 4.1 cmH_2_O (− 2.9 cmH_2_O, *p* < 0.05) and driving pressures from 18.3 ± 4.3 to 15.6 ± 3.9 cmH_2_O (− 2.7 cmH_2_O, *p* < 0.05), respectively (Table 4). Ventilatory parameters showed a trend toward baseline values over the remaining study period (Fig. 3). After reduction in ventilator settings, a lower PaO_2_ (91.8 ± 23.8 vs. 84.4 ± 18.7 mmHg, *p* < 0.05) corresponding to a lower oxygenation index (164 ± 38 vs. 151 ± 35 mmHg, *p* < 0.05) was observed (Table 4).

**Table 4:** Changes in ventilation and oxygenation parameters between baseline and after reduction in tidal volumes at 1 h

	Baseline	+ 1 h	Δ (%)	*P*-value
*V*_T_/PBW (ml/kg)	6.2 ± 0.9	5.4 ± 1.1	− 0.8 (− 12.9)	< 0.05
RMV (l/min)	9.9 ± 2.1	8.5 ± 2.1	− 1.4 (− 14.1)	< 0.05
*P*_plat_ (cmH_2_O)	30.6 ± 4.6	27.7 ± 4.1	− 2.9 (− 9.5)	< 0.05
Δ*P* (cmH_2_O)	18.3 ± 4.3	15.6 ± 3.9	− 2.7 (− 14.8)	< 0.05
PaO_2_ (mmHg)	91.8 ± 23.8	84.4 ± 18.7	− 7.4 (− 8.1)	< 0.05
*P*/*F* (mmHg)	164 ± 38	151 ± 35	− 13 (− 7.9)	< 0.05

*V*_T_ tidal volume; *PBW* predicted body weight; *RMV* respiratory minute volume; *P*_plat_ plateau pressure; Δ*P* driving pressure; *PaO*_*2*_ arterial O_2_ partial pressure; *P*/*F* oxygenation index

### Extracorporeal circuit

Out of 20 patients, 16 (80%) completed the 72-h study period. The total running time of the system was 95.8 ± 47.7 h (range 14.5 to 223.4 h). Altogether five systems were lost due to clotting, three of which were replaced and the study continued. There were no interruptions of the therapy due to patient transport. The average activated partial thromboplastin time (aPTT) was 62 ± 16 s after 24 h, 57 ± 19 s after 48 h and 59 ± 20 s after 72 h at an average continuous dose of unfractioned heparin of 1154 ± 556 IU/h, which corresponds to two times the reference range (25–34 s). The targeted blood flow rate of 400 ml/min was reached in 17/20 patients (85%). In the majority of patients (12/20), vascular access was via a jugular Shaldon catheter, whereas a femoral catheter was used in the remaining patients. In two out of three patients in whom the targeted blood flow rate could not be reached, vascular access was via a left jugular Shaldon catheter which was consequently changed. The recorded pressures in the arterial and venous line of the circuit were − 148 ± 27 mmHg and 164 ± 36 mmHg, respectively. Transmembranous pressures in the hemofilter increased continually over time from 46 ± 22 mmHg at baseline to 76 ± 21 mmHg at 72 h, whereas oxygenation performance of the membrane lung (∆PO_2_) decreased from 350 ± 62 to 311 ± 63 mmHg. Renal replacement was effected by conventional hemofiltration (CVVHF) using bicarbonate-buffered replacement fluids with an average dose of 2615 ± 470 ml/h of total effluent. Renal parameters as well as phosphate levels developed as expected, which resulted in a significant decrease in plasma creatinine (3.30 ± 2.59 vs. 1.42 ± 0.42 mg/dl) and urea (72 ± 50 vs. 31 ± 18 mg/dl) over the study period. Serum bicarbonate did not depart significantly from baseline values. A slight decrease in average thrombocyte count compared to baseline was observed after 24 h (164 ± 75 vs. 184 ± 89 nl^−1^), and thrombocytes reached baseline levels after 72 h (179 ± 94 nl^−1^).

### Systemic hemodynamics

No adverse cardiovascular effects were observed following implementation of the extracorporeal circuit. Two patients died within 24 h after inclusion due to refractory septic shock. In the remaining patients, hemodynamic improvement with markedly reduced vasopressor requirements (norepinephrine dose 3.1 ± 2.9 vs. 2.4 ± 2.0 mg/h, *p* < 0.05) and heart rate (103 ± 18 vs. 91 ± 23 bpm, *p* < 0.05) was recorded after 24 h. Hemodynamics remained stable over the further study period.

### Complications

Four patients received blood products during the study; altogether 11 units of packed red blood cells (RBC) and one unit of thrombocytes were transfused. The major part of those blood products (eight units of packed RBC and one unit of thrombocytes) was given to two patients who had previously undergone allogeneic stem cell transplantation, thus confounding transfusion requirements. No bleeding directly attributed to the extracorporeal circuit was reported and no other clinical relevant hematological abnormalities such as signs of hemolysis or hyperfibrinolysis were detected during the course of the study. No other adverse effects were reported.

## Discussion

Using a standardized protocol of ventilation based on current ARDS Network recommendations, we were able to demonstrate that the investigated combination therapy was able to ameliorate respiratory acidosis and effectively reduce the invasiveness of mechanical ventilation in hypercapnic critically ill patients while providing efficient renal replacement therapy and exhibiting a positive effect on hemodynamics in terms of vasopressor requirements. While combinations of ECCO_2_R and CRRT have previously been reported, our study provides the first description of a certified and labeled combination therapy on a commercially available organ support platform. The system was able to eliminate CO_2_ at a rate between 40 and 50 ml/min, thereby reducing arterial PCO_2_ significantly by about 10%. The additional integration of a membrane lung into a renal replacement circuit has first been described by Forster et al. [32], who were able to show a reduction in acidosis and decreased vasopressor requirements in ten hypercapnic patients. This concept was taken one step further by Allardet-Servent et al. [33], who were able to realize an ultra-protective ventilation strategy in 11 patients with ARDS using a similar combination. In both of these studies, membrane lungs with surfaces of about 0.7 m^2^ were used, which resulted in a higher CO_2_ removal rate and more pronounced correction of acidosis compared to our system, which incorporated a significantly smaller membrane lung (0.32 m^2^). As has recently been shown by Karagiannidis et al. [35], in low-flow ECCO_2_R the effectiveness of CO_2_ extraction is mainly a function of the membrane size. Whereas smaller membrane lungs may have advantages with regard to costs and likelihood of clotting, membrane size must be considered the most important limiting factor of the presented system. Another factor pertaining to combinations of ECCO_2_R and CRRT is the relative position of membrane lung and hemofilter, which may affect CO_2_ removal. In [33], the incorporation of the membrane lung downstream of the hemofilter was significantly less effective than in an upstream position. In our study, the CO_2_ removal rate of the combined system was about 5% lower than the elimination rate of the membrane lung alone, hinting at the same effect. We suggest that the substitution of bicarbonate-rich replacement fluids as a consequence of renal replacement therapy leads to an increase in the CO_2_ content of extracorporeal blood, thereby counteracting the overall effectiveness of CO_2_ removal by “loading” the blood with CO_2_. It would be interesting to investigate whether the use of citrate-based solutions which could also provide effective anticoagulation is associated with a more pronounced CO_2_ removal effect. Due to the relatively high citrate dosing requirements at the investigated blood flow (400 ml/min), no such combination is currently available.

Though the resulting drop in PaCO_2_, as is expected in a low-flow setting, is not sufficient to completely correct respiratory acidosis, implementation of the system still allowed for a significant decrease both in tidal volume (− 0.8 ml/kg) and plateau pressure (− 2.9 cmH_2_O) and in driving pressure (− 2.7 cmH_2_O). Since the study protocol did not require neuromuscular blockade to prevent spontaneous breathing and consequently only a quarter of the patients received neuromuscular blocking agents at some point during the study, there was considerable heterogeneity in response to plateau pressure reduction following implementation of ECCO_2_R with some patients counteracting the decreased inspiratory pressures by actively increasing spontaneous breathing efforts, thus mitigating the effect on tidal volume reduction.

In a recent study on the efficacy and safety of low-flow ECCO_2_R using the same platform in patients without renal failure, a more pronounced reduction in tidal volume and plateau pressure was reported [36]. In that study, all patients were paralyzed, making tidal volume reductions more easy to achieve. Also, the severity of illness was significantly different with a mean SOFA score of 9 as compared to 14 in our study. It is important to note that patients with combined respiratory and renal failure may constitute a different target group for ECCO_2_R than patients with isolated respiratory failure. In established multi-organ failure with often severe concomitant metabolic acidosis, there is typically an indication for renal replacement therapy, making the integration of an additional gas exchanger in the circuit much less invasive since vascular access is already in place. Our data show that combining ECCO_2_R with CRRT in the setting multi-organ failure, while being less effective than stand-alone therapies, can still significantly enhance lung protection and may therefore have beneficial effects. In contrast to [36], this effect can be achieved without further raising PaCO_2_, thus providing much better control of pH. Interestingly, while the additional surface of the hemofilter might be expected to activate coagulation, incidence of circuit clotting was much lower in the combination therapy than in the stand-alone procedure.

As it has been concluded by Gattinoni et al., ventilator-associated lung injury essentially results from the application of mechanical power to the lungs in order to actively eliminate CO_2_ from the circulation [37, 38]. The components of this mechanical power are tidal volume, driving pressure, PEEP, flow and respiratory rate. Any additional CO_2_ elimination is therefore capable of reducing the power applied to the lungs and consequently should attenuate ventilator-associated lung injury. This is mirrored in our study by significantly decreased tidal volumes and driving pressures. Although this rationale may seem compelling and comparable strategies have been able to show reduced systemic and pulmonary inflammation in experimental [39, 40] as well as in clinical ARDS [14, 27], to date it has not been demonstrated that ultra-protective ventilation strategies per se can improve clinically relevant patient outcomes.

Although partial extracorporeal CO_2_ elimination must therefore still be regarded as experimental at this juncture, due to its easy implementation and management, the combination of low-flow ECCO_2_R and CRRT nevertheless constitutes a safe and effective add-on therapy for ventilated patients with renal failure. Since the procedure runs on an established renal replacement platform and therefore only requires integration of a small membrane lung as well as a moderate increase in blood flow without need for specialized vascular access, the potential for complications seems low. Under systemic anticoagulation, the combined system exhibited reasonable circuit lifetimes and we observed no procedure-related bleeding or other relevant adverse events. This is in marked contrast to ECMO or even mid-flow ECCO_2_R therapies where higher blood flows can provide total CO_2_ removal but require large-bore and often multiple vascular access which is associated with significant bleeding risk and other local as well as systemic complications [12–14, 16]. We therefore conclude that combined ECCO_2_R and CRRT with the investigated system is a feasible and safe approach. However, due to the limited running time of the hemofilter for renal replacement, the system has to be discarded after 72 h, leading potentially to higher costs in prolonged treatments.

Our study has several limitations. In order to keep interference with local practice standards at the three centers at a minimum, no explicit ventilation strategy other than compliance with ARDS Network recommendations was stipulated. After the initial data collections, ventilation strategy was left to the discretion of the treating physician leading to considerable heterogeneity among the study population. With a growing number of spontaneously breathing patients over the study period, the effect of ECCO_2_R on ventilation is blurred to a considerable degree. Since neuromuscular blockade was not required, even the initial data collections may be significantly influenced by spontaneous breathing efforts. As expected in combined lung and renal failure, metabolic acidosis significantly contributes to overall acid base status. Consequently, in a number of patients, while ECCO_2_R led to a significant drop in PaCO_2_, marked overall acidosis remained, preventing a reduction in ventilator settings by the treating physician. We therefore cannot exclude that the overall effect on ventilation is confounded to some degree in this study. Furthermore, the study included only patients who already exhibited severe hypercapnia. Our data therefore allow no statement on the efficiency of the system in a normocapnic or only mildly hypercapnic environment. Due to the limited running time of the system (72 h), we also cannot provide data on long-term clinical effects.

## Conclusions

In this study, we present the first description of a combination of extracorporeal CO_2_ removal and renal replacement therapy on a commercially available organ support platform in mechanically ventilated hypercapnic patients with renal failure. The investigated combination therapy was able to ameliorate hypercapnic acidosis and allow for a decrease in ventilation pressures while providing adequate renal replacement therapy. The system was easy to implement and manage; no severe procedure-related adverse events were observed. Whether strategies aiming at correcting hypercapnia and/or providing ultra-protective ventilation are beneficial for patients with combined lung and renal failure in terms of outcome remains to be demonstrated in future investigations.

## Abbreviations

CO_2_: carbon dioxide
ARDS: acute respiratory distress syndrome
ECCO_2_R: extracorporeal CO_2_ removal
PaCO_2_: arterial CO_2_ partial pressure
PBW: predicted body weight
ECMO: extracorporeal membrane oxygenation
pECLA: pumpless extracorporeal lung assist
CRRT: continuous renal replacement therapy
aPTT: activated partial thromboplastin time
CVVHF: continuous veno-venous hemofiltration
RASS: Richmond agitation and sedation scale
*P*_plat_: plateau pressure
BMI: body mass index
SAPS: Simplified Acute Physiology Score
SOFA: Sequential Organ Failure Assessment
COPD: chronic obstructive pulmonary disease
CIHD: chronic intermittent hemodialysis
NMBA: neuromuscular blocking agent
SD: standard deviation
HCO_3_: bicarbonate
CaCO_2_: arterial CO_2_ content
SBE: standard base excess
SaO_2_: arterial oxygen saturation
*P*/*F*: oxygenation index
*V*_T_: tidal volume
RR: respiratory rate
RMV: respiratory minute ventilation
PEEP: positive end-expiratory pressure
Δ*P*: driving pressure
*C*_dyn_: dynamical compliance
HR: heart rate
MAP: mean arterial pressure
PCO_2_: CO_2_ partial pressure
PaO_2_: arterial O_2_ partial pressure
ΔPO_2_: O_2_ partial pressure difference
RBC: red blood cells
